# Predictors of tympanostomy tube extrusion time in otitis media with effusion

**DOI:** 10.15537/smj.2022.43.7.20220323

**Published:** 2022-07

**Authors:** Abdulaziz K. Alaraifi, Abdullah S. Alkhaldi, Ibrahim S. Ababtain, Fahad A. Alsaab

**Affiliations:** *From the Division of Otolaryngology - Head and Neck Surgery (Alaraifi, Alkhaldi, Alsaab), Department of Surgery, King Abdulaziz Medical City, Ministry of National Guard Health Affairs, and from College of Medicine (Ababtain), King Saud bin Abdulaziz University for Health Sciences, Riyadh, Kingdom of Saudi Arabia*.

**Keywords:** otitis media, recurrence, predictors, tympanostomy, ventilation tube, extrusion

## Abstract

**Objectives:**

To investigate the impact and predictors of tympanostomy tube (TT) extrusion.

**Methods:**

A retrospective study on 258 ears underwent TT insertion during 2016-2018 at King Abdullah Specialized Children’s Hospital, Riyadh, Saudi Arabia. Patients were followed for 36-48 months postoperatively to detect the recurrence rate. The sample was divided into 2 groups based on extrusion time and were compared to determine the predictors of TT extrusion.

**Results:**

Otitis media with effusion (OME) recurrence after TT insertion was detected in 28.7%. A shorter TT extrusion time was associated with a higher recurrence (p=0.002). Small TTs increased the probability of early TT extrusion (odds ratio = 5.144; 95% confidence interval: [1.602-16.519]).

**Conclusion:**

More than one-fourth of the patients who underwent TT insertion for OME developed recurrence. Tympanostomy tube extrusion earlier than 12 months was associated with a higher recurrence rate. Small TTs increased the probability of early TT extrusion.

**O**titis media with effusion (OME) is the most common cause of pediatric hearing loss.^1,2^ It is defined as the accumulation of fluids behind an intact tympanic membrane, with the absence of the signs and symptoms of acute infection.^3^ Most OME episodes resolve spontaneously without intervention. However, around 10% of cases require myringotomy with tympanostomy tube (TT) insertion due to failure of conservative treatment.^3,4^ Tympanostomy tube insertion is one of the most commonly carried out procedures in children with OME, aiming to release the middle ear effusion (MEE) and ventilate the middle ear. The complications of this procedure include otorrhea, myringosclerosis, atrophy of the tympanic membrane, formation of granulation tissue, and early extrusion of TT.^3^

Otitis media with effusion has a considerable recurrence rate post myringotomy with TT insertion, ranging between 19.9-53.2%.^5-10^ Tympanostomy tube extrusion time is an essential factor that influences the recurrence rate of OME, where a longer extrusion time is associated with a lower recurrence rate.^11^ Variable factors were reported in the literature to affect the TT extrusion.^12-14^ These include patient-related factors (such as age and gender), surgery-related factors (such as tube size, tube shape, and concurrent surgery), and disease-related factors (such as adenoid size, characteristics of effusion, recurrent infections postoperatively, and tympanometry findings). This study investigates the TT extrusion time with its impact and predictors to provide more input to the current literature.

## Methods

A retrospective cohort study was carried out at King Abdullah Specialized Children’s Hospital in Riyadh, Saudi Arabia. All pediatric patients (aged 14-year-old or younger) who were diagnosed with OME and underwent myringotomy with TT insertion between 2016-2018 were included in the study. The patients who have head and neck anomalies were excluded from the study.

The diagnosis of OME existed based on clinical and tympanometric findings. The patients with persistent OME for more than 3 months with a documented hearing loss underwent myringotomy with TT insertion. Shah-type fluoroplastics TTs of 2 sizes were used; regular TTs (1.14 cm inner diameter with 1.58 cm interflange distance) and small TTs (0.76 cm inner diameter with 1 cm interflange distance). Small tubes were used in patients with narrow or tortuous external auditory canals, while all the other patients received regular TTs. Patients were followed for 36-48 months postoperatively in 2-3 months intervals to detect the recurrence rate.

The data collection sheet included baseline clinical and demographic characteristics (such as age, gender, and body mass index [BMI]), history of otolaryngology-related conditions (such as recurrent acute otitis media and adenoid hypertrophy), OME-related details (such as unilateral versus bilateral, tympanometry findings), surgery-related details (characteristics of intraoperative effusion, tube size, and TT extrusion time), concurrent surgery (such as adenoidectomy or tonsillectomy), and recurrence status. The data were extracted from medical charts and Hospital Health Information System.

### Statistical analysis

The data were analyzed using Statistical Package for the Social Sciences, version 25.0 (IBM Corp., Armonk, NY, USA). The categorical data were summarized and reported as frequencies and proportions, while the continuous variables were summarized and reported as means and standard deviations (SD). The recurrence rate was estimated for all study samples, and based on extrusion time in 3 subgroups (1-6, 7-12, and >12 months). Then, the study sample was divided into 2 groups based on TT extrusion time (≤12 and >12 months). The 2 groups were compared using univariable and multivariable regression analysis to determine the predictors of TT extrusion time.

## Results

The study included 258 ears from 144 patients. The mean age of the patients was 5.19±4.0 years old, with males dominating the females (62.5% versus 37.5%). Bilateral OME constituted 89.6% of the cases, and more than two-thirds of the patients had adenoid hypertrophy (70.1%). The proportions of patients who underwent adenoidectomy were 63.2% and tonsillectomy were 33.3%.

**Figure 1:**
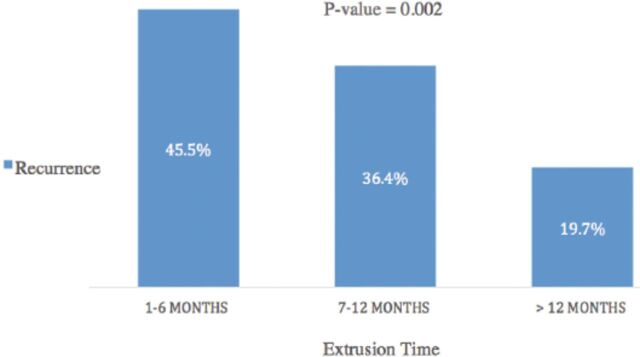
- The recurrence rate of otitis media with effusion based on tympanostomy tube extrusion time.

Otitis media with effusion recurrence after myringotomy with TT insertion was detected in 74 (28.7%) ears. Tympanostomy tube lasted more than 12 months in more than half (53.1%) of the cases, with a mean extrusion time of 13.96±6.5 months (Table 1). A higher recurrence rate was significantly associated with a shorter TT extrusion time (*p*=0.002). Figure 1 illustrates the relationship between TT extrusion time and recurrence rate.

**Table 1:** - Tympanostomy tube extrusion time in all operated ears (N=258).

Variables	n (%)
***TT extrusion time, mean±SD***	13.96±6.5)
1-6 months	33 (12.8)
7-12 months	88 (34.1)
>12 months	137 (53.1)
***Tube diameter***	
1.14 mm	237 (91.9)
0.76 mm	21 (8.1)

Values are presented as a number and precentage (%). TT: tympanostomy tube, SD: standard deviation

The tube size was the only significant predictor of early TT extrusion (Table 2). Small TTs (0.76 mm) lasted less than 12 months in most cases (81%), compared to regular tubes, which lasted less than 12 months in 43.9% of the cases. Small TTs increased the probability of early TT extrusion by 5.14 times, as illustrated in Table 3.

**Table 2:** - Predictors of tympanostomy tube early extrusion.

Variables	TT extrusion time ≤12 months (n=121)	TT extrusion time >12 months (n=137)	*P*-value
***Age***			
≤5 years	86 (51.2)	82 (48.8)	0.059
>5 years	35 (38.9)	55 (61.1)	
***Gender***			
Male	81 (49.1)	84 (50.9)	
Female	40 (43.0)	53 (57.0)	0.347
BMI, mean±SD	17.03±4.9	16.86±4.4	0.510
***OME***			
Unilateral	11 (52.4)	10 (47.6)	0.599
Bilateral	110 (46.4)	127 (53.6)	
***Concurrent adenoidectomy***			
Yes	73 (44.0)	93 (56.0)	0.206
No	48 (52.2)	44 (47.8)	
***Adenoid size***			
Grade 1	36 (46.8)	41 (53.2)	0.21
Grade 2	53 (57.0)	40 (43.0)	
Grade 3	32 (36.4)	56 (63.6)	
***Tympanometry***			
Type B	114 (46.9)	129 (53.1)	0.946
Type C	7 (46.7)	8 (53.3)	
***MEE***			
Negative	12 (60.0)	8 (40.0)	0.474
Serous	54 (45.8)	64 (54.2)	
Thick	55 (45.8)	65 (54.2)	
***Tube diameter***			
1.14	104 (43.9)	133 (56.1)	0.01^*^
0.76	17 (81.0)	4 (19.0)	

Values are presented as a number and precentage (%).

* Significant at *p*<0.05 level, TT: tympanostomy tube, BMI: body mass index, OME: otitis media with effusion, MEE: middle ear effusion, SD: standard deviation

**Table 3:** - Predictors of early tympanostomy tube extrusion in logistic regression analysis.

Variables	Odd ratio	*P-*value	95% LCI	95% UCI
Small tube diameter	5.144	0.006^^*^^	1.602	16.519
Age (≤5 years)	1.596	0.095	0.922	2.763
Male gender	1.377	0.245	0.803	2.360
Recurrent AOM	1.345	0.304	0.764	2.368
Concurrent adenoidectomy	0.761	0.536	0.320	1.808
Adenoid hypertrophy	1.064	0.816	0.630	1.797
Tympanometry type B	0.617	0.270	0.262	1.454
Absence of MEE	1.075	0.736	0.708	1.631

* Significant at *p*<0.05 level. LCI: lower confidence interval, UCI: upper confidence interval, AOM: acute otitis media, MEE: middle ear effusion

## Discussion

The majority of OME cases resolve spontaneously within 3 months. Myringotomy with TT insertion is carried out for patients with persistent MEE despite watchful waiting, which is seen in around 10% of cases.^3,4^ The insertion of TTs helps improve hearing function and patients’ overall quality of life. However, the recurrence rate following TT insertion is still high. The recurrence rate in our study is 29%, which falls in the range of reported rates in the literature.^5-9^ The reported predictors of OME recurrence following TT insertion included patient-related factors (such as age, gender, atopy, BMI, and craniofacial anomalies), surgery-related factors (such as tube insertion, tube size, tube shape, extrusion time, and adenoidectomy), and disease-related factors (such as adenoid size, characteristics of effusion, recurrent infections, and tympanometry findings).^5,7^

Tympanostomy tubes are usually extruded spontaneously from the tympanic membrane within 6-24 months without the need for manipulation or removal. The mean extrusion time in our study was 13.96±6.5 months. Early extrusion of TTs was reported as a factor that increased the recurrence rate in multiple studies.^11,15^ The present study showed a higher recurrence rate in ears with a shorter TT extrusion time (Figure 1). The ears that had TT for more than 12 months have a recurrence rate of nearly half of those which lasted less than 12 months. This finding was consistent with the other studies that showed a higher recurrence rate once that extrusion time was less than 12 months.^16,17^

Multiple studies investigated the factors that influence the timing of TT extrusion, but the results are variable. The present study found that the size of TTs was the only predictor of TT extrusion time. Small TTs extruded prematurely more frequently compared to regular tubes. The finding correlated with Lin et al^15^, who recommended avoiding small TTs in children to prevent premature extrusion. Moreover, Paparella et al^18^ recommended choosing TT size based on short-term or long-term purposes. Our study was confined to Shah-type fluoroplastics tubes; however, the shape and material of the tubes were reported to affect its extrusion time. Short-term TT (such as Shepard, Armstrong, Shah, Sheehy, Reuter Bobbin, Donaldson, and Paparella type I) are associated with a shorter extrusion time and higher recurrence rate but fewer overall complications. On the other hand, long-term TTs (such as Paparella type II, Butterfly, Per-Lee, and Goode T-tubes) have a longer extrusion time and lower recurrence rate but a higher rate of complications. These complications include otorrhea, persistent perforation, myringosclerosis, atrophy of the tympanic membrane, formation of granulation tissue, and cholesteatoma.^17,19^ Moreover, Kim et al^20^ showed that thermoplastic elastomer TTs have a longer extrusion time than silicone TTs.

Adenoid hypertrophy and patient’s age were reported to influence the extrusion time in the literature. Younger children were more likely to have early extrusion than older patients in multiple studies.^11,21,22^ This relationship could be explained by the decreasing incidence of OME and the growth of Eustachian tube (ET) as patients get older. However, our study and others showed no significant association between age and extrusion time.^12^ Ahn et al^21^ demonstrated that concurrent adenoidectomy decreases the need for revision TT insertion as it can relieve ET’s mechanical obstruction and reduce the pathogens load in the nasopharynx. However, our study showed no significant association between adenoid size and adenoidectomy with TT extrusion time, supporting the findings of Song et al.^14^

The viscosity of MEE, which varies from thick mucus fluid to serous secretion, was reported to affect the recurrence rate of OME.^23^ The MEE was divided into thick, serous, and negative intraoperative effusion in the present study. Our study showed no significant association between MEE characteristics and TT extrusion time, supporting the conclusion of Gibb et al^12^ and Maw et al.^24^ Moreover, there was no significant association between preoperative tympanometry findings and TT extrusion in the present study. Song et al^14^ and Leopold et al^22^ found that revision TT insertion was associated with a longer extrusion time. This association could be explained by the postoperative changes in the tympanic membrane resulting from the surgery. Previous surgery could result in scarring and atrophy of the tympanic membrane due to losing the middle fibrous layer during the healing process, affecting the tympanic membrane’s elasticity.^19^ Our study did not examine this relationship as it was confined to primary surgeries.

### Study limitations

Our sample was collected from one tertiary healthcare center and was confined to one shape and material of TTs. This limitation can affect the generalizability of the study. However, the present study investigated the predictors of TT extrusion time in a large sample with a long-term follow-up period. Additional prospective randomized clinical trials with a larger sample are needed to validate predictors of TT extrusion time.

In conclusion, more than one-fourth of the patients who underwent TT insertion for OME developed recurrence within 3-4 years. Most TTs lasted in the tympanic membrane for more than 12 months; however, tube extrusion earlier than 12 months was associated with a higher recurrence rate. Small TTs increased the probability of early TT extrusion.
